# A Unique Case of Mitral Valve Dehiscence Post-mitral Valve Repair Diagnosed Using 3D Transesophageal Imaging

**DOI:** 10.7759/cureus.6541

**Published:** 2020-01-02

**Authors:** Raj D Patel, Mansoor Ahmad, Harshavardhan Ghadiam, Tinoy Kizhakekuttu

**Affiliations:** 1 Cardiology, University of Illinois College of Medicine at Peoria, Peoria, USA; 2 Internal Medicine, University of Illinois College of Medicine at Peoria, Peoria, USA

**Keywords:** mitral valve dehiscence.

## Abstract

This is an interesting cardiovascular imaging case of a 70-year-old male who presented with heart failure symptoms after recent mitral valve repair with Carpentier-Edwards ring. Ring dehiscence was noted on transesophageal echocardiographic imaging which aided in guiding clinical and surgical courses.

## Introduction

Mitral valve annuloplasty ring dehiscence with recurrent mitral regurgitation represents a challenge. If left untreated it has high mortality without surgical correction or valve replacement [1].

## Case presentation

We present the case of a 70-year-old Caucasian male with a known history of atrial fibrillation, essential hypertension, hyperlipidemia prior stroke, and chronic dilated cardiomyopathy with an ejection fraction of 50%-55%. Over the course of approximately 10 years, the patient developed progressive posteriorly directed mitral regurgitation secondary to left ventricular dilatation. The patient was followed with yearly echocardiograms; in early 2018, he began developing signs and symptoms of heart failure with progressive decline. He was unable to perform activities of daily living without becoming profoundly short of breath. Repeat echocardiogram in 2018 showed a progression of mitral regurgitation with indices in the severe range. The patient underwent a transesophageal echocardiogram (TEE) for further evaluation of mitral regurgitation and was found to have a mitral valve effective regurgitant orifice (ERO) of 0.53 cm2, a regurgitant volume of 74 mL by proximal isovelocity surface area (PISA), and systolic reversal of pulmonary vein flow. Ejection fraction on the TEE was noted to be 45%. He was promptly referred to outpatient cardiothoracic surgery for evaluation and was deemed an ideal candidate for mitral valve repair. The patient concurrently had moderate tricuspid regurgitation. A right heart catheterization revealed normal pulmonary artery (PA) and pulmonary capillary wedge pressure (PCWP). He had minimal, nonocclusive coronary artery disease. In September of 2018, the patient underwent successful mitral valve repair with a 34 mm Carpentier-Edwards Physio IITM (Edwards Lifesciences Corp., Irvine, CA) annuloplasty ring. Concurrently, the patient underwent tricuspid annuloplasty with a 34 mm Edwards MC3TM (Edwards Lifesciences Corp., Irvine, CA) ring. Due to long-standing atrial fibrillation, the patient also underwent MAZE procedure with left atrial appendage ligation.

**Figure 1:**
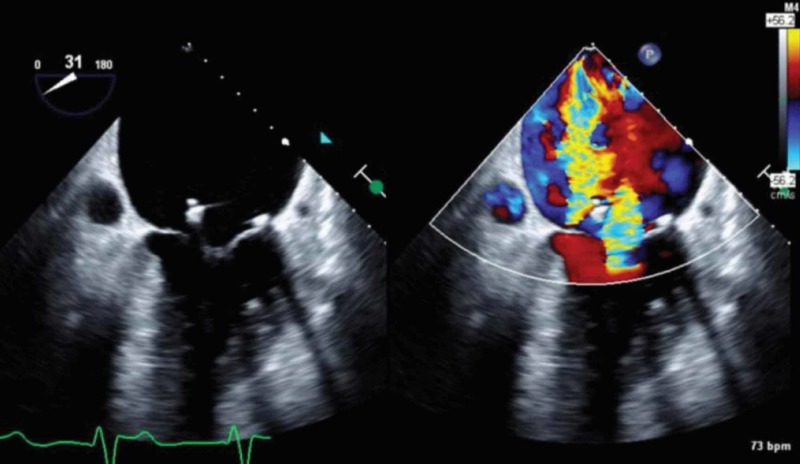
Severe mitral regurgitation noted on 2D TEE image (Left) of the mitral valve at 30-degree mid-esophageal view. Color Doppler with regurgitant jet (Right). TEE, transesophageal echocardiogram

**Figure 2:**
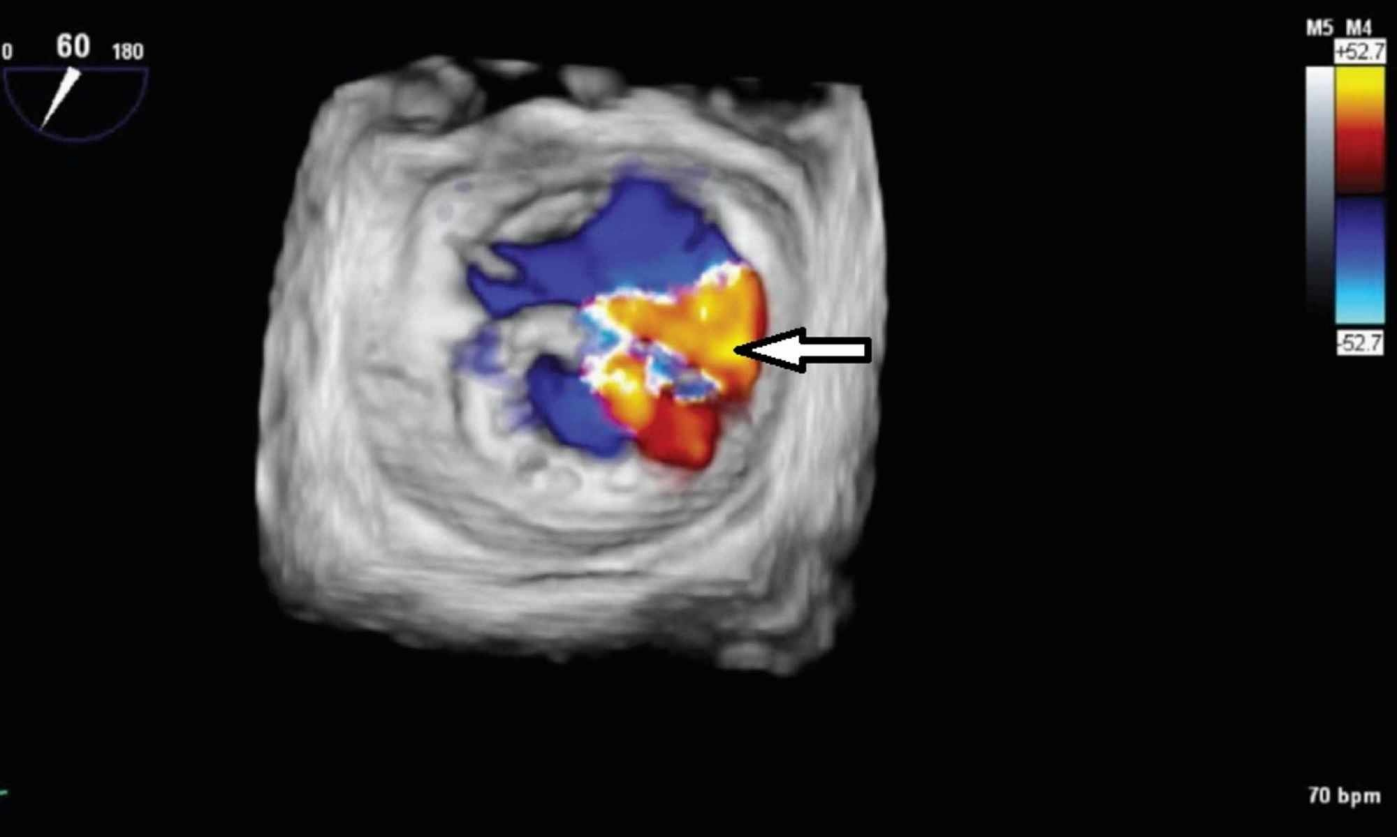
Severe mitral regurgitation (white arrow) and ring dehiscence noted on 3D Color Doppler TEE imaging of the mitral valve in en face view. TEE, transesophageal echocardiogram

**Figure 3:**
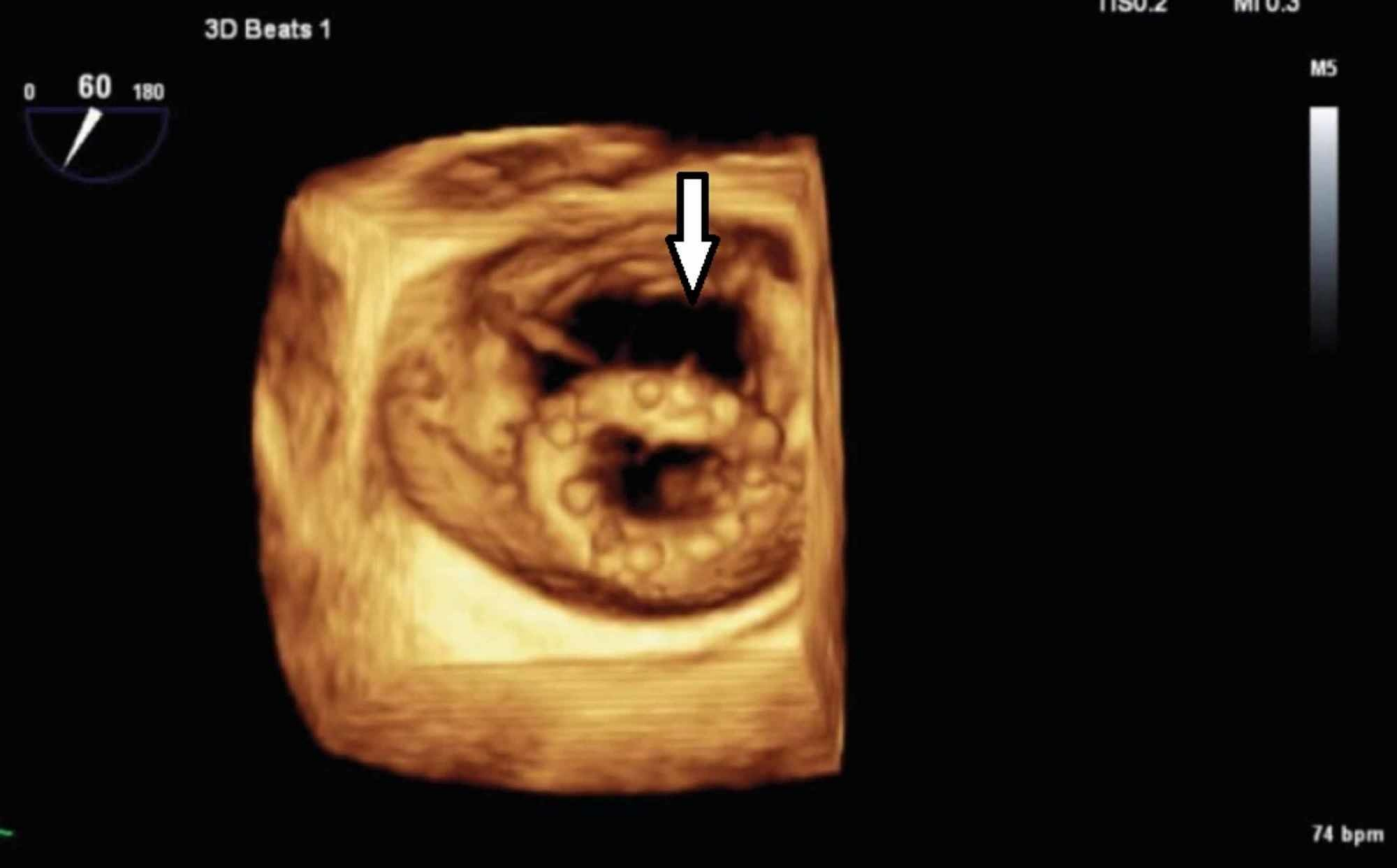
3D TEE imaging without color revealing significant ring dehiscence (white arrow) along the anterior mitral valve. TEE, transesophageal echocardiogram

The patient suffered no immediate postoperative complications and was discharged home in stable condition. However, the patient continued to have progressive decline over the next several months. He complained of significant shortness of breath on exertion, lower extremity edema, and overall malaise/weakness. Oral furosemide was initiated in the outpatient setting. He eventually presented to the hospital in December 2018 with New York Heart Association (NYHA) Class III heart failure symptoms. After the initiation of intravenous furosemide for heart failure, he underwent a TEE to evaluate the mitral and tricuspid valve. He was found to have severe mitral regurgitation (Figure 1) with two regurgitant jets secondary to anterior mitral ring dehiscence. He also had moderate tricuspid regurgitation. Neither valve had evidence of vegetations. The 3D images were constructed with TEE (Figures 2-3).

Cardiovascular surgery was consulted. The decision was made to proceed with mitral valve replacement and tricuspid valve repair. During surgery, the patient was found to have dehiscence starting in an area of calcification at posterior scallop (P3 segment) of mitral valve. The ring was removed and replaced with a 33 mm Magna Ease Mitral Valve. The tricuspid valve showed partial dehiscence in the region of the septal leaflet which was then revised using a pledgeted stitch to anchor the M3 ring in place. The patient had postoperative tachy-brady syndrome and underwent placement of a MicraTM (Edwards Lifesciences Corp., Irvine, CA) permanent pacemaker. After an otherwise noncomplicated hospital course, the patient was discharged home in stable condition.

## Discussion

Compared with 2D echocardiography, current 3D probes, due to the high element count (>3,000), deliver the high sampling rates necessary to achieve clinically useful realtime 3D imaging with high image resolution and satisfactory frame rates. Imaging in 3D essentially involves the acquisition of a volume data set over one or more heartbeats, depending on volume size. However, a limitation of the current technology is that as the volume of interest increases in size, image quality is compromised in terms of spatial and temporal resolution.

Mitral valve annuloplasty ring dehiscence leading to symptomatic mitral valve regurgitation is a challenging problem [1]. Annuloplasty ring dehiscence could be complete or partial [2]. Ring dehiscence is most common in the P3 segment of the mitral valve (68%), compared with P1 and P2 segments [1]. This particular case was diagnosed as early valve dehiscence (<1-year) of the P3 segment. Compared with 2D echocardiography, 3D imaging is preferred as it can better define the dehiscence of mitral valve annuloplasty, with severity and location [3-5]. Our case showed mitral valve regurgitation on 2D imaging, however, valve dehiscence could not be defined unless 3D imaging was done (Figures 2-3). The 3D imaging holds a significant place in diagnosing valve dehiscence, as mortality is reduced dramatically after reoperation [1].

## Conclusions

Transesophageal echocardiogram is the preferred modality to evaluate the mitral valve anatomy, especially before and after the procedures involving the mitral valve. Recent advancements in 3D probe technology have enabled clinicians to evaluate the structure of native and prosthetic valve for valve pathologies. Compared with traditional 2D imaging, 3D imaging is the preferred modality to diagnose mitral valve dehiscence. It also helps define the severity and location of dehiscence better than 2D imaging modalities.
